# Examining Cannabis, Tobacco, and Vaping Discourse on Reddit: An Exploratory Approach Using Natural Language Processing

**DOI:** 10.3389/fpubh.2021.738513

**Published:** 2022-01-05

**Authors:** Ryzen Benson, Mengke Hu, Annie T. Chen, Shu-Hong Zhu, Mike Conway

**Affiliations:** ^1^Department of Biomedical Informatics, University of Utah, Salt Lake City, UT, United States; ^2^Department of Biomedical Informatics and Medical Education, University of Washington School of Medicine, Seattle, WA, United States; ^3^Moores Cancer Center, University of California, San Diego, La Jolla, CA, United States

**Keywords:** marijuana, tobacco, smoking, electronic cigarettes, social media, natural language processing, Reddit, infodemiology

## Abstract

**Background:** Perceptions of tobacco, cannabis, and electronic nicotine delivery systems (ENDS) are continually evolving in the United States. Exploring these characteristics through user generated text sources may provide novel insights into product use behavior that are challenging to identify using survey-based methods. The objective of this study was to compare the topics frequently discussed among Reddit members in cannabis, tobacco, and ENDS-specific subreddits.

**Methods:** We collected 643,070 posts on the social media site Reddit between January 2013 and December 2018. We developed and validated an annotation scheme, achieving a high level of agreement among annotators. We then manually coded a subset of 2,630 posts for their content with relation to experiences and use of the three products of interest, and further developed word cloud representations of the words contained in these posts. Finally, we applied Latent Dirichlet Allocation (LDA) topic modeling to the 643,070 posts to identify emerging themes related to cannabis, tobacco, and ENDS products being discussed on Reddit.

**Results:** Our manual annotation process yielded 2,148 (81.6%) posts that contained a mention(s) of either cannabis, tobacco, or ENDS with 1,537 (71.5%) of these posts mentioning cannabis, 421 (19.5%) mentioning ENDS, and 264 (12.2%) mentioning tobacco. In cannabis-specific subreddits, personal experiences with cannabis, cannabis legislation, health effects of cannabis use, methods and forms of cannabis, and the cultivation of cannabis were commonly discussed topics. The discussion in tobacco-specific subreddits often focused on the discussion of brands and types of combustible tobacco, as well as smoking cessation experiences and advice. In ENDS-specific subreddits, topics often included ENDS accessories and parts, flavors and nicotine solutions, procurement of ENDS, and the use of ENDS for smoking cessation.

**Conclusion:** Our findings highlight the posting and participation patterns of Reddit members in cannabis, tobacco, and ENDS-specific subreddits and provide novel insights into aspects of personal use regarding these products. These findings complement epidemiologic study designs and highlight the potential of using specific subreddits to explore personal experiences with cannabis, ENDS, and tobacco products.

## Introduction

Prior work has explored the broad facets of tobacco, electronic nicotine delivery systems (ENDS), and cannabis. This existing work has focused on various aspects of product use including the use, dual use (i.e., recent use of two product types), co-use (i.e., the simultaneous use of two product types), and user opinions of these different products (1–4). Furthermore, survey data has suggested that individuals frequently co-use these products, warranting the study of these products together rather than separately (5). Using consumer-generated data—in this context, textual data derived from social media services like Twitter and Reddit—continues to gain traction as a method to generate new insights into product use behavior. Using such approaches to study tobacco, ENDS, and cannabis could provide novel firsthand accounts on the use of these products and serve to complement existing survey-based approaches to study their use.

### Tobacco Use in the United States

Since the release of the first US Surgeon General's report in 1964, the prevalence of combustible tobacco use has substantially declined in the US (6). Although smoking prevalence continues to decline, an estimated 16.7% of the US adult population still currently uses combustible tobacco products, and smoking remains the leading cause of preventable death in the US (6, 7). Combustible tobacco use is associated with a multitude of comorbidities, including but not limited to cardiovascular disease, chronic obstructive pulmonary disease (COPD), and numerous cancers (8). While these health detriments have increased the desire among smokers to quit, the 2015 National Health Interview Survey (NHIS) showed that only 7.4% of past year smoking cessation attempts were successful (9). Combustible tobacco use is more prevalent among males vs. females (20.1 and 13.6% respectively) and individuals between 25 and 44 years of age (7). Additionally, in the US smoking is more common among adults with lower educational attainment, lower-income status, and American Indian/Alaska Native individuals^1^. Developing targeted tobacco cessation strategies among these individuals is a critical step in obtaining the goals outlined by Healthy People 2030 to reduce the prevalence of smoking in the U.S.^2^ and, in turn, mortality due to smoking.

### ENDS Use in the United States

ENDS or electronic cigarettes, are devices in which a nicotine solution that is often artificially flavored, is heated into a vapor that is then inhaled to simulate the act of smoking. ENDS devices include but are not limited to vape pens (pen-shaped nicotine vaporizers), mods (modifiable nicotine vaporizers), pod-mods (ENDS devices with disposable nicotine pods), and vaporizers (devices with refillable tanks for nicotine solutions)^3^. These devices differ mechanistically and in nicotine delivery, but were initially developed as a cessation aide that resembles the feel and experience of smoking cigarettes (10). Studies have found that the use of ENDS among adult smokers increased the rate of quit attempts and helped those who did attempt to quit achieve a higher rate of sustained abstinence (11–14). In recent years, ENDS use has continually increased, especially among adolescents (15). In 2019, it was estimated that ~4.5% of US adults used an ENDS device, compared to 10.5 and 27.5% of US middle school and high school students, respectively (7, 16). This increase in popularity is largely attributable to the emergence of pod mods, ENDS devices with sleek designs that mimic the nicotinic delivery of combustible cigarettes (17, 18). Although adults commonly use ENDS devices to aid in smoking cessation (6), studies have suggested that adolescents and young adults may be using ENDS devices recreationally rather than to aid smoking cessation (19, 20). Further, a recent study demonstrated that from 2014 to 2018, the adolescent age of initiation for ENDS devices continued to decrease (21), meaning that adolescents were beginning ENDS use at younger ages. These use patterns have sparked great concern about the potential for youth users to develop nicotine dependence, subsequent nicotine addiction, and a later transition to combustible cigarettes (22–24). In addition to the concerns of youth nicotine exposure, the long-term effects of ENDS use have yet to be ascertained, an outcome that can only be observed prospectively with future study.

### Cannabis Use in the United States

Cannabis (marijuana) has long been stigmatized as an illicit drug in the US, despite the demonstrated health benefits associated with its use, including pain relief, chronic disease management, and stress reduction (25). However, there is also substantial evidence regarding the potential harms associated with cannabis use. Some of these detrimental health effects include neurological structure alterations, the onset of mental health disorders, cannabis dependency development, and the onset of various chronic conditions (26). From 2002 to 2014, significant increases in cannabis use and initiation were observed among US adults (27). Moreover, following the November 2020 US elections, 19 states and two US territories have now legalized recreational cannabis use and 35 states have legalized medical cannabis^4^,^5^, where states with legalized cannabis tend to show a higher prevalence of use than states without legalized recreational cannabis (28, 29). In addition to the changing legislation surrounding medical and recreational cannabis, the forms and potency of cannabis available on the market continue to change as well. Tetrahydrocannabinol (THC) is the primary psychoactive cannabinoid found in cannabis and the determining compound of cannabis potency. Lab tests of cannabis seized by the Drug Enforcement Administration (DEA) showed that the average THC content had increased 4-fold from 3% in 1980 to 12% in 2012 (30). This high potency, accompanied by the various methods of ingestion (i.e., bud/flower, edibles, topicals, dab pens, extracts) (31), as well as the conflicting evidence regarding the health effects of its use, highlight the critical need for further study of user habits and perceptions of cannabis.

### Using Social Media in Public Health Research

For over a decade, the popularity of social media sites continues to rise, as do the functionalities of these sites (e.g., Instagram for posting pictures, Twitter for tweeting short posts, TikTok for posting short videos). Thanks to the development of social media-specific application programming interfaces (APIs), publicly available data posted on these sites can be collected for analysis by end users. As a result, public health researchers have leveraged this available data to study numerous aspects of public health. The research questions that can be explored are largely dependent on the specific site used to obtain data for analysis, as certain social media sites are better suited for specific research questions due to the structure of the website, community posting guidelines put in place, and limits posed by post structure.

Reddit^6^ is a popular social media site in which members under a self-chosen username post discussions within a subreddit (i.e., a forum centered around a common theme such as smoking cessation or cannabis use). As a result of this anonymity, members often discuss sensitive and oftentimes stigmatized health behaviors and conditions (32). Furthermore, unlike other social media sites such as Twitter and Facebook, Reddit posts are often more verbose, and since they are aggregated into subreddits centered around common themes of interest, the development of Reddit-specific APIs have resulted in an increasing body of literature leveraging Reddit to study aspects of substance use. Reddit has been used to study the effects of the COVID-19 pandemic on opioid use patterns and disruptions to treatment (33, 34), emerging forms of cannabis discussed in subreddits (35), potential adverse health effects associated with specific JUUL pod flavors (36), and to study linguistic patterns characteristic of alcohol and tobacco abstinence (37), to name a few. Further, we recently published results of natural language processing (NLP) pipelines developed to identify the prevalence of tobacco, cannabis, and ENDS mentions within tobacco, cannabis, and ENDS-related subreddits (38). However, that prior study was primarily concerned with the computational identification of tobacco, cannabis and ENDS mentions in Reddit posts, hence we did not further analyze these posts for the topics discussed within. Therefore, there remains need to investigate the topics of discussion frequent to tobacco, cannabis, and ENDS-centric subreddits.

To address this gap, our study uses qualitative and computational methods to explore member experiences and discussions in eight different subreddits related to cannabis, ENDS, and tobacco product use and to identify differentiating characteristics of these subreddits for assisting with the future study of these products on Reddit. We first explore member-specific experiences as well as general discussion of the three product types by manually annotating posts made in these eight subreddits. Second, we apply Latent Dirichlet Allocation (LDA) topic modeling to our macro-corpus (i.e., posts made in these subreddits over a 5-year span) to determine emerging themes in these posts through an unsupervised manner. Last, we create word clouds for each product (e.g., cannabis, tobacco, and ENDS) based on the vocabulary of our annotated corpus to compare the broad themes discussed in our annotated corpus vs. those topics identified by topic modeling. By understanding the discourse related to these three product types on Reddit, our work serves to identify the use patterns, behaviors, and common topics associated with these products, complementing existing methods for studying differences and similarities in product use behavior.

## Methods

### Data Collection

**Figure 1:**
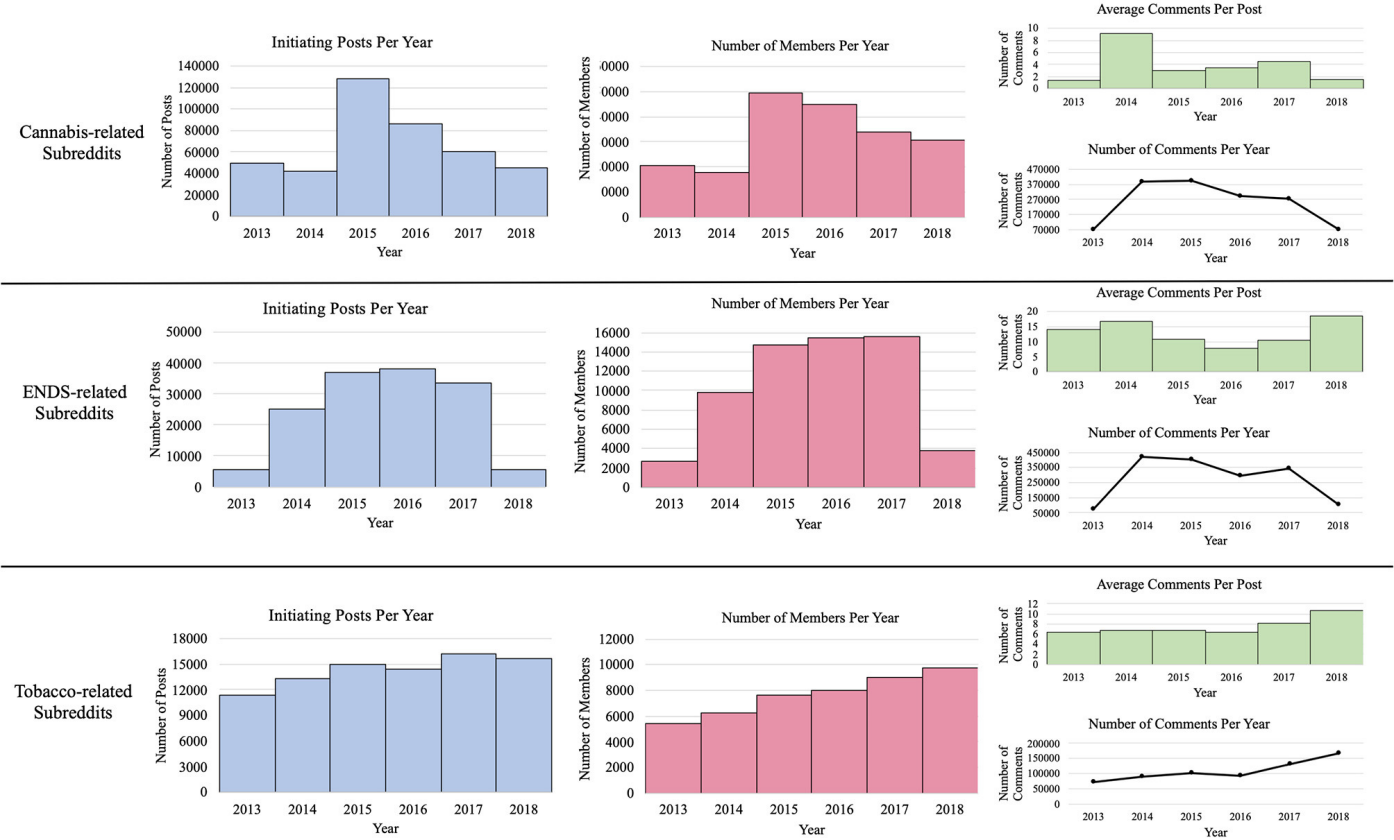
Member and post statistics for macro-corpus, stratified by product type.

**Figure 2:**
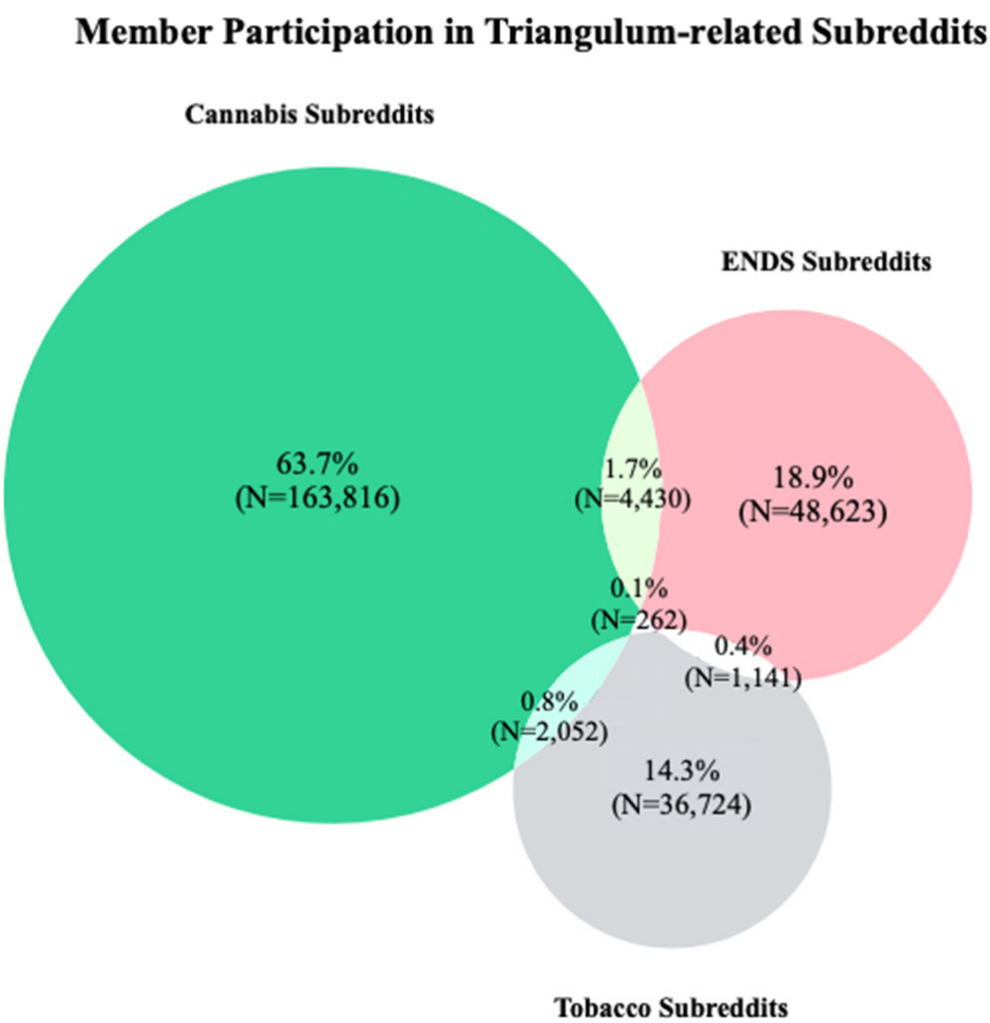
Representation of the frequency of members posting in cannabis, tobacco, and ENDS-specific subreddits in our macro-corpus.

There are numerous subreddits focused on cannabis, ENDS, and tobacco, all of which serving a different purpose and with varying levels of member participation. Therefore, we selected a subset of subreddits with at least 50,000 members, demonstrating adequate member engagement within the subreddit, and providing a sufficient sample size to carry out our research objectives. Using the pushshift.io API (39, 40), we downloaded all available data from eight subreddits related to cannabis, ENDS, and tobacco between January 2013 to December 2018. These subreddits consisted of r/Vaping, r/electronic_cigarette, r/Vaping101, r/weed, r/trees, r/Marijuana, r/Cigarettes and r/stopsmoking. Prior work has observed frequent topic drift—a phenomenon where conversation in forums drift from the topic of interest to a different topic, particularly among online forums dedicated to health-related subjects (41). To mitigate topic drift, and in an attempt to observe member-specific experiences and discussion, we only analyzed initiating posts in this study and did not evaluate subsequent comments. Our resulting macro-corpus consisted of 643,070 initiating posts. Figure 1 presents a breakdown of the posts, subsequent comments, and members contained in our macro-corpus. We then stratified the macro-corpus by substance into cannabis-specific subreddits (i.e., r/Marijuana, r/weed, r/trees), tobacco-specific subreddits (i.e., r/stopsmoking, r/Cigarettes), and ENDS-specific subreddits (r/Vaping101, r/Vaping, r/electronic_cigarette). Figure 2 provides the frequency of members participating in subreddits based on product type.

### Annotation Scheme Development

Before analyzing our Reddit posts, we developed an annotation scheme to capture the various attributes and themes related to Reddit member experiences of ENDS, tobacco, and cannabis. To develop, refine, and evaluate the annotation scheme, we randomly selected a subset of 950 initiating posts from our macro-corpus. Based on prior work (42, 43), we developed an initial list of cannabis, ENDS, and tobacco-related keywords (e.g., tobacco, electronic cigarette, marijuana). As the brand names and terms used to describe tobacco products and cannabis are frequently evolving (35, 44), we used the Gensim Python library (45) to train a neural network-based algorithm, Word2Vec, on the entirety of the macro-corpus to identify additional keywords synonymous or plesionymous (i.e., close to synonymous) to our initial keywords. To ensure that we developed an annotation scheme capturing the attributes of interest (e.g., firsthand use of combustible tobacco, historical cannabis use by someone else) for our study, we filtered posts that did not contain at least one of these keywords of interest. The resulting 280 cannabis, ENDS, and tobacco-related posts were then divided into seven batches for interrater agreement and annotation scheme development. Using the eHost annotation tool (46), authors MH, RB, AC, and MC annotated each batch of posts according to the corresponding version of the annotation scheme. Following each round of annotation, any discrepancies between annotators were discussed, resolved, and an adjudicated annotation set was created. After the seven annotation rounds, the interrater agreement reached an F-score of 0.83, a measure used as a surrogate for Cohen's Kappa and indicating a strong level of agreement among annotators (47–49). Our final annotation scheme can be found in the Supplementary Material.

### Manual Annotation

**Figure 3:**
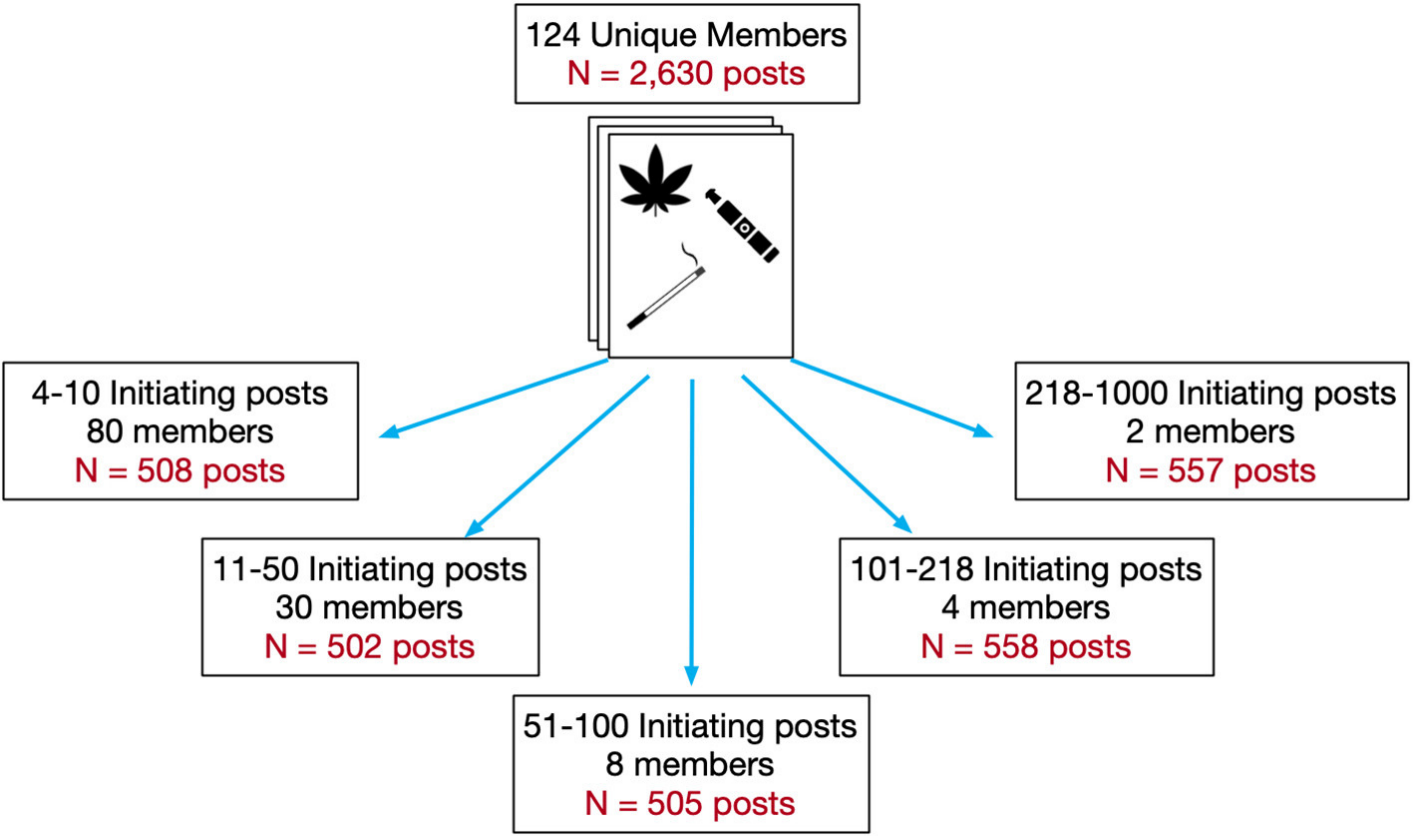
Member and post selection for manual annotation.

Having developed and evaluated our annotation scheme, we identified a subset of posts for manual annotation. Due to the observation that some members created initiating posts more frequently than others, and to ensure diversity in the posting patterns represented, we stratified our members into five bins according to the frequency of their initiating posts and selected a random sample of members from each bin. As seen in Figure 3, 80 members created between 4 and 10 initiating posts, 30 members created between 11 and 50 initiating posts, eight members created between 51 and 100 initiating posts, four members created between 100 and 218 initiating posts, and two members created between 218 and 1,000 initiating posts. Altogether, our sample comprised of 124 Reddit members and their 2,630 initiating posts from the macro-corpus. Author RB annotated the majority of these posts according to the annotation scheme, while author MC annotated a small subset of the posts to ensure the quality of the agreement was maintained. Posts may have contained one or more attributes from our annotation scheme; therefore, annotations are not necessarily mutually exclusive. The resulting proportions and frequencies from our manual annotation can be observed in Table 1.

**Table 1:** Frequencies and percentages of posts from the manual annotation.

	**r/trees *N* = 658**	**r/weed *N* = 2**	**r/Marijuana *N* = 875**	**r/stopsmoking *N* = 166**	**r/Cigarettes *N* = 37**	**r/Vaping *N* = 343**	**r/electronic_cigarette *N* = 67**
**Experiencer**						
First person experience	285 (43.3)	1 (0.5)	10 (1.1)	122 (73.4)	16 (43.2)	205 (59.7)	42 (62.6)
Experience other	20 (3.0)	0 (0.0)	0 (0.0)	12 (7.2)	0 (0.0)	7 (2.0)	1 (1.4)
General discussion	486 (73.8)	2 (100.0)	872 (99.6)	130 (78.3)	32 (86.4)	225 (65.5)	63 (94.0)
**Temporality**						
Historical	14 (2.1)	0 (0.0)	1 (0.1)	46 (27.7)	1 (2.7)	3 (0.8)	4 (5.9)
Present	286 (43.4)	1 (0.5)	10 (1.1)	102 (61.4)	15 (40.5)	202 (58.8)	42 (62.6)
Future	6 (0.9)	0 (0.0)	1 (0.1)	2 (1.2)	0 (0.0)	17 (4.9)	1 (1.4)
**Product**						
Marijuana	655 (99.5)	2 (100.0)	874 (99.8)	2 (1.2)	1 (2.7)	3 (0.8)	0 (0.0)
Combustible tobacco	33 (5.0)	1 (0.5)	6 (0.6)	165 (99.3)	37 (100.0)	14 (4.0)	8 (11.9)
ENDS	5 (0.7)	0 (0.0)	0 (0.0)	8 (4.8)	0 (0.0)	341 (99.4)	67 (100.0)

*Annotations are not necessarily mutually exclusive*.

### Data Pre-processing

To pre-process our data for computational analyses, we first converted all of the Reddit posts to lower case. Using the Natural Language Toolkit (NLTK) (50), a widely used Python module for text analysis, we then split (i.e., tokenized) each post into individual word tokens. Lastly, we iterated through each token and removed any stop words. Stop words (e.g., “the,” “is,” “what”) are words that are common (51) but may not necessarily contribute semantic meaning to a corpus.

### Latent Dirichlet Allocation and Word Clouds

While our manual annotation provides a comprehensive view of member experiences with cannabis, ENDS, and tobacco on Reddit, manually annotating our entire macro-corpus (N = 643,070) is not feasible. Consequently, topic modeling proves to be an invaluable method for studying commonly discussed topics within a large textual corpus. Latent Dirichlet Allocation (LDA) is one of these unsupervised machine learning techniques used to identify topics discussed in a corpus. Topics in an LDA model are represented by the grouping of words that are similar or co-occur throughout the corpus (51–53). LDA has been used to study several public health topics, including but not limited to infectious diseases, obesity, vaccinations, and health communications (32, 54–56), and has been shown effective in identifying salient topics within unstructured text sources such as social media. To develop our LDA models, we again used the Gensim library—as it supports various topic modeling techniques (45) and pyLDAvis to assist with the interpretation of the most salient terms for each topic (57). Then using our stratified macro-corpus (i.e., cannabis-specific subreddits, ENDS-specific subreddits, and tobacco-specific subreddits), we developed three distinct LDA models, one for each product type. We manually varied LDA model hyperparameters (i.e., number of iterations and batch size to train the models) to determine the optimal number of topics (k) to identify themes frequent to each product type. We then manually inspected the resulting terms with these varying k values to observe coherence between and ultimately determine the relevant number of k topics, as well as the best label that encompasses the observed terms. Lastly, using our annotated corpus, we developed a word cloud for each product type using the WordCloud Python library (58). These analyses together, allowed us to compare commonly discussed topics in both our annotated corpus and the larger macro-corpus.

### Ethical Considerations

This study was determined to be exempt from review by the University of Utah Institutional Review Board (IRB#00076188). To protect Reddit member privacy, we have refrained from including usernames in this paper. Further, all quotations used are synthesized from multiple examples.

## Results

### Macro-Corpus

As seen in Figure 1, we saw a large increase in member participation and the number of posts made in cannabis-specific subreddits in 2015, followed by decreases through 2018. In ENDS-specific subreddits, we observed annual increases in the number of posts within these subreddits between 2013 and 2016, followed by a slight decrease in volume in 2017 and a drastic decrease in 2018, whereas tobacco-specific subreddits saw continual increases in posts made and member participation per year between 2013 and 2018. In total, the 643,070 posts in our macro-corpus were posted by 257,048 unique members across all eight subreddits of interest. Some members deactivated their Reddit accounts between the date of the post and when we collected the Reddit posts. Therefore, those usernames are not available and are referred to as “[Deleted]” by the API.

As shown in Figure 2, 63.7% of members (*N* = 163,816) posted exclusively in cannabis-specific subreddits, 18.8% of members (*N* = 48,623) posted exclusively in ENDS-specific subreddits, and 14.3% of members (*N* = 36,724) posted exclusively in tobacco-specific subreddits. The remaining ~3% of members posted in multiple product-specific subreddits. We observed 4,430 members posting in both cannabis and ENDS-specific subreddits, 2,052 members posting in both cannabis and tobacco-specific subreddits, 1,141 members posting in both ENDS and tobacco-specific subreddits, and 262 members who posted in cannabis, tobacco, and ENDS-specific subreddits.

### Manual Annotation

The complete results of our manual annotation can be seen in Table 1. Of the 2,630 posts that we manually annotated, 2,148 (81.6%) posts contained at least one mention of either cannabis, ENDS, or tobacco products. 1,537 (71.5%) of these posts mentioned cannabis, 421 (19.5%) posts mentioned ENDS, and 264 (12.2%) posts mentioned tobacco. Only three posts explicitly mentioned the dual use of cannabis, ENDS, and tobacco products, and 11 posts explicitly mentioned transitions between these products. Two of the three posts mentioning dual use were made by one member. In these posts, the member explicitly mentioned their personal experiences with these products and stated current use of all three products. Upon closer examination of the posts containing transitions between products, six posts were made in the r/Vaping subreddit, three were made in the r/stopsmoking subreddit, and two posts were made in the r/electronic_cigarette subreddit. All 11 posts were related to past, ongoing, or planned smoking cessation attempts or associated relapse events, and none of these posts mentioned cannabis. Four posts discussed relapse from ENDS devices to combustible cigarettes, while three posts documented ongoing smoking cessation attempts using ENDS devices. Additionally, one post discussed a failed past cessation attempt using ENDS, two posts discussed contemplating the use of ENDS to facilitate a future cessation attempt, and one post discussed a relapse from ENDS devices to cigarettes and then subsequently reinitiating ENDS use.

Within r/Marijuana, 16 members posted general discussions (e.g., news stories, questions, non-personal experiences with cannabis), and three members posted self-experiences with cannabis compared to r/trees where 70 members posted self-experiences with cannabis, and 65 members posted general discussions, suggesting that members are more likely to post personal experiences with cannabis in the r/trees subreddit compared to r/Marijuana. Of the 311 posts containing mentions of experiences with cannabis, 16 (0.5%) posts mentioned former use, 299 (96.1%) posts mentioned current use, and 7 (0.2%) posts mentioned potential use in the future. Of the 185 posts containing mentions of experiences with tobacco, 53 (28.6%) posts mentioned former use or past cessation attempts, 158 (85.4%) posts mentioned current use or current cessation attempts, and 7 (3.7%) posts mentioned potential use or contemplation of potential cessation attempts in the future. Of the 261 posts containing mentions of ENDS experiences, 13 (4.9%) posts mentioned former use, 252 (96.5%) posts mentioned current use, and 19 (7.2%) posts mentioned potential use in the future.

### Latent Dirichlet Allocation and Word Cloud Analysis

**Figure 4:**
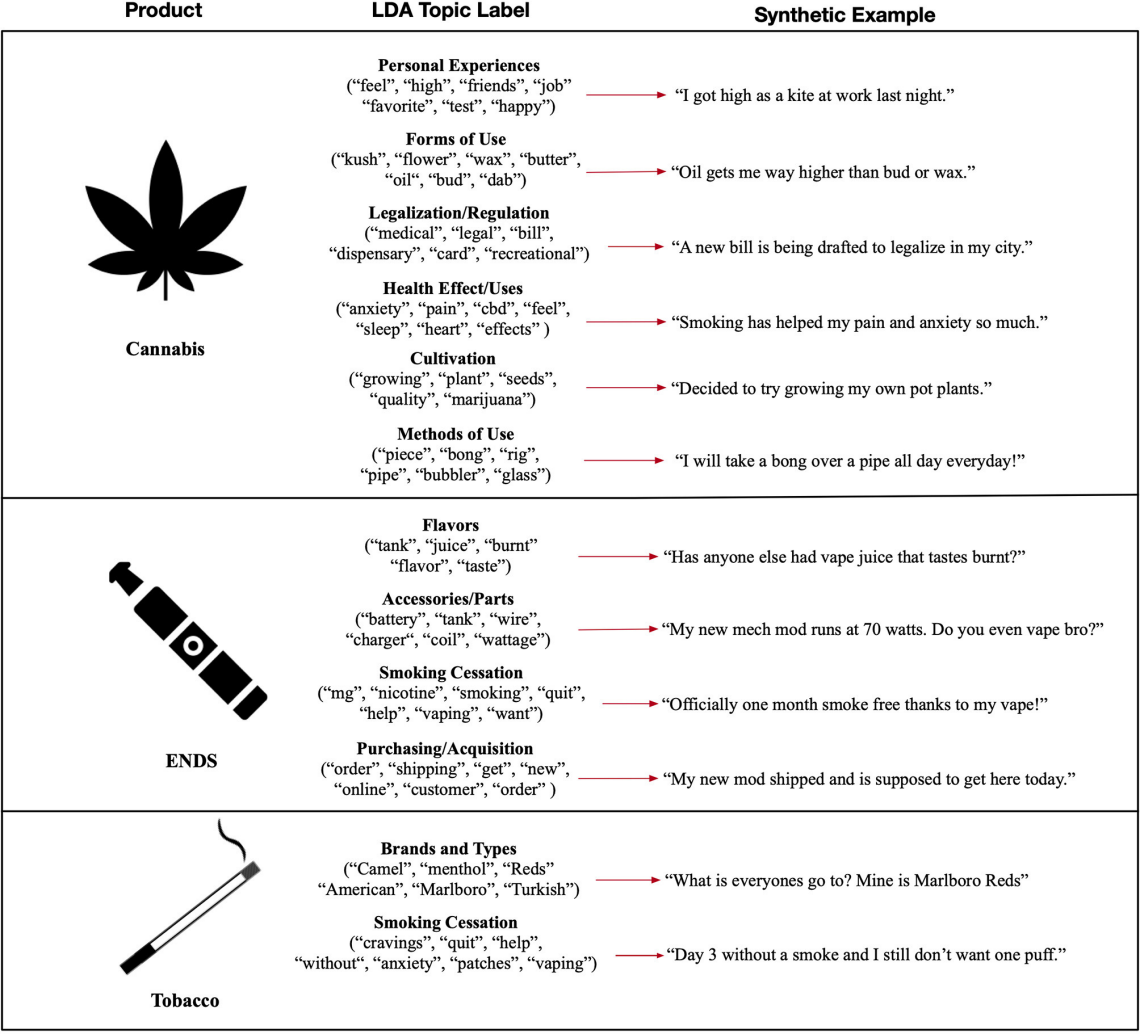
Resulting topics from our LDA analysis, accompanied by common terms observed in the topic and synthetic textual examples.

In the LDA models applied to our macro-corpus, we observed six frequently discussed topics in cannabis-specific subreddits, four frequent topics in ENDS-specific subreddits, and two common topics in tobacco-specific subreddits. Cannabis-related topics included the legalization of medicinal and recreational cannabis, experiences with and recreational use of cannabis, the methods and forms of cannabis, health effects and uses of cannabis, and the cultivation of cannabis plants. ENDS-specific subreddits often held discussions of different flavors and nicotine solutions, accessories and parts, procurement of ENDS devices, and the use of ENDS for smoking cessation. And tobacco-specific subreddits often contained discussions of different brands and types of combustible tobacco and current, past, and planned smoking cessation attempts. Figure 4 provides the common terms used to derive each topic label from our LDA analysis and synthetic textual examples of these topics.

**Figure 5:**
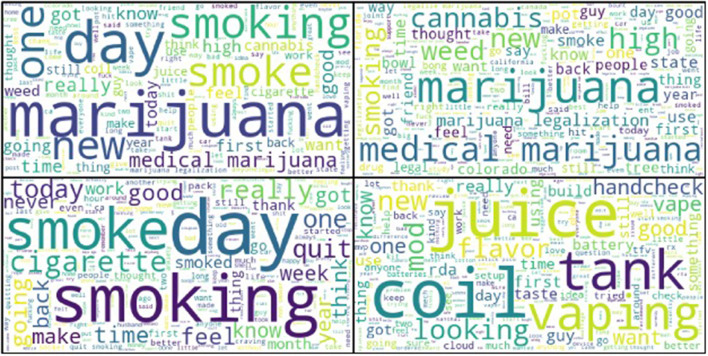
Word cloud representations of our annotated corpus of 2,148 Reddit posts. Top left: all posts, top right: cannabis-related posts, bottom left: tobacco-related posts, bottom right: ENDS-related posts.

The observed topics from the LDA analysis of our macro-corpus closely mimic the terms from the word clouds of our annotated posts, as seen in Figure 5. In the word clouds developed from our annotated corpus, the cannabis-specific word cloud consisted of terms including “cannabis,” “high,” “legalization,” and “medical.” In the tobacco-specific word cloud, we observed terms such as “smoke,” “quit,” and terms that may suggest temporality components of smoking cessation such as “time,” “today,” “month,” and “year.” Furthermore, in the ENDS-specific word cloud, we observed terms such as “flavor,” “tank,” “mod,” and “juice.”

## Discussion

### Principal Findings

Reddit is a popular social media platform in which members post content in communities (subreddits) centered around everyday topics. Reddit posts are typically more verbose than other social media sites (e.g., Twitter, Instagram) and have numerous subreddits dedicated to the discussion of various aspects related to cannabis, tobacco, and ENDS products. Leveraging this structure, we studied eight subreddits focused on cannabis, tobacco, and ENDS, identifying emerging themes and analyzing how these themes differ between product types.

In cannabis-specific subreddits, we observed a large increase in the number of posts and the number of Reddit members within these subreddits in 2015. This observation is likely a result of the increase in proposed cannabis legislation in the mid 2010's, a controversial topic of debate throughout the United States^7^. Our topic analysis of these subreddits also supports this finding as cannabis policy was one of the emerging themes discussed throughout our macro-corpus of posts. In addition to legislation and policy, members in cannabis-specific subreddits frequently talked of personal experiences with cannabis, the health effects of cannabis use, methods and forms of cannabis, and the cultivation of cannabis. Upon manual annotation and a closer examination of posts containing cannabis-related discussion, the subreddits r/weed and r/Marijuana appear to harbor more general discussion of cannabis, including discussion related to legalization and regulation, a finding also observed in prior work (32). The vast majority of posts containing personal experiences of cannabis were found in the subreddit r/trees. Within these experiential cannabis-related posts, members often talk about methods of consuming cannabis, experiences while under the influence, stories of cannabis use, and were typically centered around present use of cannabis. While previous studies have used other social media sites to explore the use of cannabis (59, 60), and a prior study compared cannabis use as discussed on one subreddit (35), no study, to the best of our knowledge, has studied aspects of cannabis use across multiple subreddits. Consequently, these findings are significant contributions—demonstrating the potential of Reddit data to explore opinions and use patterns of cannabis while also providing guidance as to which subreddits are best for studying personal use experiences versus general discussion. Based on our findings, future work that seeks to leverage Reddit for studying cannabis use and perceptions should do so using the r/trees subreddit.

From 2013 to 2018, we observed continual increases in the number of posts made and members participating in tobacco-specific subreddits. This sustained volume of posts within these subreddits may be a result of the observed increase in desire among smokers to quit in recent years (61), but may be an artifact of increasing usage of Reddit since 2012^8^. These patterns are consistent with our topic modeling analysis in which we observed smoking cessation frequently being discussed throughout posts made in tobacco-specific subreddits. In addition to aspects of cessation, we frequently observed discussions among current smokers regarding their favorite brands and types of combustible tobacco (e.g., Marlboro, unfiltered). These discussions were mostly housed in r/Cigarettes, a subreddit dedicated to discussions of cigarettes among smokers. Conversely, posts in r/stopsmoking were typically from current smokers attempting or contemplating a quit attempt, individuals experiencing relapse from a smoking cessation attempt, or individuals who successfully quit smoking and sustained their abstinence. In this subreddit, members documented their quit journey, including withdrawal symptoms and side effects, while also asking for advice to assist in their quit attempts. These findings reinforce the work of Chen et al., in which the investigators evaluated thematic elements expressed in the stopsmoking subreddit (62).

From 2013 to 2017, the frequency of Reddit members posting in ENDS-specific subreddits increased. These observed increases in ENDS-related posts may be a result of the increasing popularity of ENDS devices in the mid 2010's but may also reflect the increase in Reddit traffic since 2012, as stated prior. Pew research surveys show that 22% of Reddit members are young adults between the ages of 18 and 29^9^, this in conjunction with the increase of members in ENDS-specific subreddits may reflect the findings of Dai and Leventhal that reported increases in current and daily ENDS use among young adult populations between 2014 and 2018 (63). However, we observed drastic decreases in both the number of members and number of posts within ENDS-specific subreddits in 2018. This observation contradicts survey findings that the prevalence of ENDS use among US adults in 2018 had reached a watermark high of 7.6% over the previous 5 years (63). Most posts made in these subreddits focused on nicotine flavors, accessories, procurement of ENDS devices, as well as smoking cessation using these devices. Reddit members in the r/Vaping and r/electronic_cigarette subreddits often posted about their use patterns and experiences with ENDS devices. Consequently, member-specific participation in ENDS-related subreddits, as well as their participation in separate health-related subreddits over time (e.g., r/Health), may provide insight and generate hypotheses into the potential health effects attributable to prolonged ENDS use, and serves as a potential avenue for future computational epidemiology research.

As the scope of our manual annotation (i.e., characterizing product mentions of cannabis, tobacco, and ENDS) and our topic modeling differed, we developed word clouds in an attempt to broadly compare the similarity of discourse between posts contained in our annotated corpus and the larger macro-corpus. We found that the frequently observed terms in our annotated corpus closely resembled those topics discovered in our topic modeling, and therefore we hypothesize that many of the characteristics seen in our annotated corpus (e.g., members frequently discussing present cannabis use patterns, discussion of present smoking cessation attempts) may be frequently discussed throughout the entire macro-corpus, providing a potential avenue for future work.

In our manual annotation, we only observed three explicitly mentioned instances of product dual use. This is likely an artifact of our sampling strategy as we only annotated posts from 124 specific members where dual use was evidently uncommon. We anticipated more instances of product dual use than we observed in our manual annotation, as a substantial body of epidemiologic studies have demonstrated frequent use of multiple product types (1–4), and through the observation that many members posted in multiple subreddits as seen in Figure 2. Though we were not able to ascertain frequent dual use within this study, Reddit members may disclose these habits within individual posts, outside of the relatively small sample size of annotated posts reported in this study. Few posts explicitly discussed transitions between cannabis, ENDS, and tobacco. And of the posts that did mention transitions, all of said posts discussed transitions between ENDS and combustible tobacco use, with no posts mentioning transitions to or from cannabis. These posts frequently discussed current smoking cessation attempts facilitated by ENDS, in addition to relapse events from ENDS devices to combustible tobacco. These findings showed that Reddit members in our annotated corpus seldom talked about transitions between these products, and when they did, they were often centered around usage in smoking cessation attempts. However, these patterns of transition may be more common in the larger macro-corpus upon further analysis.

Our study has some limitations to be considered. First, our analysis was limited to posts from eight popular subreddits focused on cannabis, ENDS, and tobacco. However, the content posted in these subreddits may contain posts discussing non-related topics (i.e., topics not related to cannabis, ENDS, tobacco). Although our work focused on larger subreddits, additional subreddits also discuss cannabis, ENDS, and tobacco products, such as r/leaves (i.e., discussion of cessation from THC-containing products). Future work may look to build on our analysis and include these additional subreddits closely related to cannabis, ENDS, and tobacco products (e.g., r/cannabis, r/vaporents, r/DIY_eJuice, r/cigars, r/hookah). Second, while we manually annotated 2,630 Reddit posts, these posts only encompassed 124 Reddit members as a result of our sampling strategy. Although we observed use patterns comparable to prior epidemiologic studies, we cannot ascertain the geographic locations of Reddit members, nor can we assume that posts, opinions, and use patterns observed in our analysis are representative of all cannabis, ENDS, and tobacco members. Therefore, we cannot generalize these findings to the general population. Furthermore, this small sample of members may not encompass the true representation of our attributes of interest as seen on Reddit. Third, social media sites often fall victim to a phenomenon known as the 90-9-1 principle (64), where the large majority of members on a social media site just observe posts and do not contribute, a small proportion of members contribute sparingly, and a small number of members who contribute the majority of posts. Consequently, any findings resulting from social media data are difficult to generalize to the general population. Fourth, as seen in Figure 1, there was an observed decrease in posts collected during 2018. We expected to see continual increases in posts made in 2018, as a result, these observed decreases may be a result of decreases in the number of posts retrievable by the API, or discrepancies with parsing posts collected by the API. Finally, the posts in our analysis were collected between 2013 and 2018. With the consistently changing landscape regarding ENDS devices, cannabis legislation, and combustible tobacco use, our results may differ upon the analysis of more recent posts from these subreddits.

## Conclusion

In conclusion, our study compared aspects of cannabis, ENDS, and tobacco use across multiple subreddits on the social media site Reddit. We found that Reddit posts provide firsthand accounts of cannabis, ENDS, and tobacco use and can complement findings derived from traditional survey-based approaches. In subreddits dedicated to cannabis, members frequently discussed personal experiences, methods and forms of use, legislation and policy, the associated health effects of its use, as well as the cultivation of cannabis plants. In tobacco specific subreddits, members often talked about ENDS devices, documented their smoking cessation attempts, and discussed brands and types of cigarettes. Further, ENDS-specific subreddits were often focused on parts and accessories, flavors and nicotine solutions, the procurement of ENDS devices, and discussion of smoking cessation using ENDS devices. Upon closer examination of these posts, the r/trees, r/stopsmoking, r/Vaping, and r/electronic_cigarette subreddits were more commonly used to discuss personal experiences with cannabis, ENDS, and tobacco. Future computational research should look to expand upon the manual annotation scheme presented and develop models to classify personal experiences from general product mentions for a more focused analysis on cannabis, ENDS, and tobacco use as presented on Reddit.

## Data Availability Statement

The datasets presented in this article are not readily available because this dataset contains social media usernames which if shared, may result in the identification of those individuals. Consequently, this data is not to be shared. Requests to access the datasets should be directed to ryzen.benson@utah.edu.

## Author Contributions

RB and MC conceived the study idea with support from S-HZ. MC, MH, AC, and RB developed the annotation scheme presented and carried out the manual annotation of the data. RB developed the computational models and analyzed the data. RB also led the manuscript writing with support from MC, AC, and S-HZ. All authors contributed to the article and approved the submitted version.

## Funding

The research reported in this publication was partially supported by the National Institute on Drug Abuse of the National Institutes of Health under award number R21DA043775. The content is solely the responsibility of the authors and does not necessarily represent the official views of the National Institutes of Health or the National Institute on Drug Abuse. This study was also partially supported by grant number T15LM007124 from the National Library of Medicine. The content is solely the responsibility of the authors and does not necessarily represent the official views of the National Library of Medicine or the National Institutes of Health.

## Conflict of Interest

The authors declare that the research was conducted in the absence of any commercial or financial relationships that could be construed as a potential conflict of interest.

## Publisher's Note

All claims expressed in this article are solely those of the authors and do not necessarily represent those of their affiliated organizations, or those of the publisher, the editors and the reviewers. Any product that may be evaluated in this article, or claim that may be made by its manufacturer, is not guaranteed or endorsed by the publisher.

## Footnotes

^1^ https://cancercontrol.cancer.gov/brp/tcrb/monographs/monograph-22

^2^ https://health.gov/healthypeople/objectives-and-data/browse-objectives/tobacco-use

^3^ https://www.cdc.gov/tobacco/basic_information/e-cigarettes/pdfs/ecigarette-or-vaping-products-visual-dictionary-508.pdf

^4^ https://www.usnews.com/news/best-states/articles/where-is-marijuana-legal-a-guide-to-marijuana-legalization

^5^ https://www.medicinenet.com/how_many_states_legalized_medical_marijuana_2021/article.htm

^6^ http://www.reddit.com

^7^ https://www.pewresearch.org/politics/2015/04/14/in-debate-over-legalizing-marijuana-disagreement-over-drugs-dangers/

^8^ https://backlinko.com/reddit-users

^9^ https://www.statista.com/statistics/261766/share-of-us-internet-users-who-use-reddit-by-age-group/
