# Supplementary figures and images for: Examining Cannabis, Tobacco, and Vaping Discourse on Reddit: An Exploratory Approach Using Natural Language Processing
Source: Front Public Health. 2022 Jan 5;9:738513. doi: 10.3389/fpubh.2021.738513 (PMC8766503; doi:10.3389/fpubh.2021.738513)

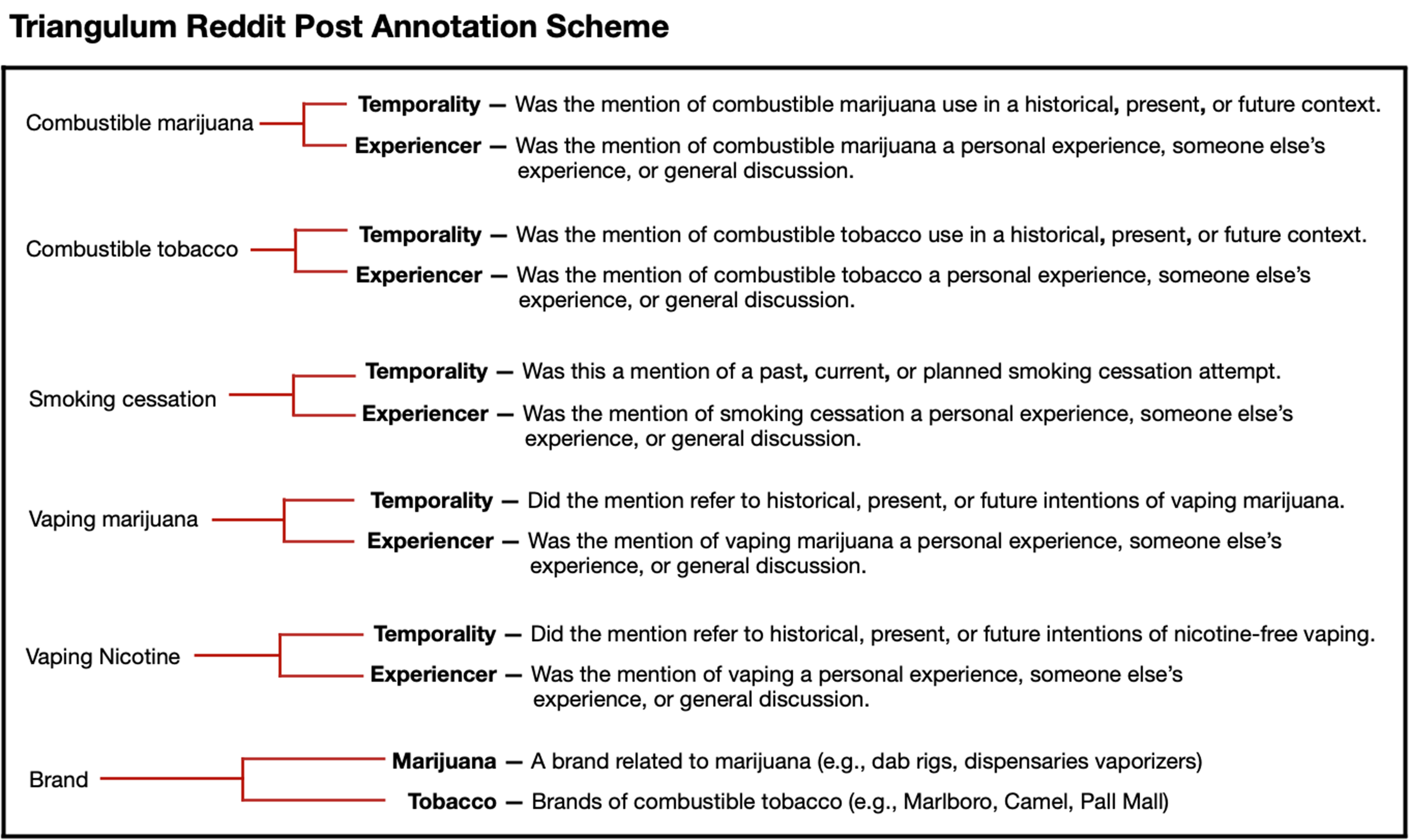

Supplement: Supplementary file 1 [file Image_1.png]
